# Effects of an Intensive Lifestyle Intervention to Treat Overweight/Obese Children and Adolescents

**DOI:** 10.1155/2017/8573725

**Published:** 2017-06-05

**Authors:** Claudia Ranucci, Roberto Pippi, Livia Buratta, Cristina Aiello, Vincenza Gianfredi, Natalia Piana, Elisa Reginato, Alberto Tirimagni, Emanuele Chiodini, Emilia Sbroma Tomaro, Alessio Gili, Pierpaolo De Feo, Carmine Fanelli, Claudia Mazzeschi

**Affiliations:** ^1^Centro Universitario di Ricerca Interdipartimentale Attività Motoria (C.U.R.I.A.Mo.), Healthy Lifestyle Institute, University of Perugia, Perugia, Italy; ^2^School of Specialization in Hygiene and Public Health, Department of Experimental Medicine, University of Perugia, Perugia, Italy

## Abstract

**Objective:**

The purpose of the present study is to examine the effects of a multidisciplinary lifestyle intervention to treat overweight/obese children and adolescents. The main outcome was cardiometabolic risk based on the waist-to-height ratio (WHTR) measurement. Secondary outcomes were (1) changes in body composition; (2) adherence to a Mediterranean diet; and (3) physical performance.

**Method:**

The study involved 74 overweight/obese children or adolescents. The intervention was multidisciplinary including nutrition, exercise, and psychological aspects based on a family-based approach; it was delivered for six months for children and three months for adolescents. Before and after the intervention, several anthropometric measures (height, body weight, body mass index or BMI, waist circumference, and body composition), cardiometabolic risk index (waist-to-height ratio or WHTR), and nutrition habits of the participants and their families were evaluated. In addition, a set of functional motor fitness tests was performed to evaluate physical performance measures.

**Results:**

After the intervention both children and adolescents showed a significant reduction in body weight, BMI, waist circumference, fat mass, and WHTR index and an improvement of fat-free mass, adherence to the Mediterranean diet, and physical fitness performance.

**Conclusion:**

A family-based multidisciplinary approach is effective in the short term in ameliorating the health status, the nutrition habits, and physical performance in children and adolescents.

## 1. Introduction

The lack of physical activity (PA) [1] or low levels of PA and sedentary habits with overeating/unhealthy eating are the most causes of the increase of obesity [2–4].

Obesity is a multifactorial disease, the product of the complex interaction of genetic, hormonal, physical, nutritional, social, and environmental factors [5]. Overweight and obesity are growing in childhood and adolescence all over the world.* In 2014 *the World Health Organization (WHO) estimated more than 41 million overweight children under the age of five [6]. European data show that the prevalence of overweight ranged from 18% to 57% among boys and from 18% to 50% among girls; 6−31% of boys and 5−21% of girls are obese [7]. Childhood obesity is a risk factor for adult noncommunicable diseases (NCDs) and represents a health care cost for society [8]. For these reasons childhood obesity is one of the most serious public health problems in our time and a new challenge [9].

A lot of studies show that sedentary behaviors independent of physical activity levels are associated with increased risk of all-cause mortality and psychological problems [10, 11].

Therefore, the promotion of physical activity among children and adolescents is considered a strategic way to tackle childhood obesity [12], physical activity habits developing in early life and persisting into adulthood [13, 14].

A decreased adherence to the Mediterranean diet in childhood may be related to an increase in chronic noncommunicable diseases [15]. Furthermore studies suggest a favourable role of Mediterranean diet on BMI and metabolic syndrome components like lipids profile and insulin resistance. Most children living in Mediterranean countries showed low adherence to a Mediterranean-like diet. Therefore, improving their eating habits should be an objective for the prevention and treatment of pediatric overweight and obesity [16, 17].

The prevention and the treatment of childhood obesity is complex and requires a multicomponent approach involving the family and addressing individual and social aspects [18], focusing not only on physical activity but also on nutrition and enhancing motivation toward a healthy lifestyle [19–22].

The family-based approach was defined as the “gold standard” treatment [23, 24]. As suggested by Kitzman and Beech [25] the use of a family-oriented approach to pediatric treatment of obesity can be defined as the active involvement of parents in the treatment of overweight and obesity in children. The parents' active involvement allows acting on the various multiple factors affecting childhood obesity disease. Evidence suggests that the adherence of the entire family to an intervention program represents an important predictor of the intervention success [26, 27].

Parents and their support play a key role in increasing the physical activity level of their children and in avoiding the adoption of sedentary lifestyle. They could achieve these aims through a change in the home environment, decreasing their children's screen and sitting times [28, 29].

The EPODE Umbria Region Obesity Intervention Study (EUROBIS) is an innovative program to prevent and treat childhood obesity [30] based on Ensemble Prévenons l'Obésité des Enfants (EPODE) strategy [31] to improve lifestyle habits through microenvironmental changes combined with a clinical lifestyle intervention tailored for children and adolescents, which is based on the experience of the C.U.R.I.A.Mo. model, already experimented for adult obesity [32]. Our clinical lifestyle intervention for children and adolescents has been structured as a multicomponent family-based approach taking into account and measuring the key aspects of healthy lifestyles and has been explained trough a nutritional, exercise, and psychological intervention. There are already encouraging results that structured lifestyle intervention programs for children with overweight/obesity can lead to improvements in body composition and cardiometabolic outcomes [33]. Despite this evidence additional research is needed to test the efficacy of a stepped care model for children-adolescents affected by overweight/obesity [22, 34, 35]. To our knowledge there is a lack of a reproducible and standardized clinical model of intervention [36–38]; the implementation of multicomponent programs involving the family and working on individual and sociodemographic aspects [39] through group treatment [40, 41] is recommended.

The present study reports the first results of an original clinical lifestyle intervention with a family-based approach, for children (aged 5–12 years) and adolescents (aged 13–17 years) with overweight or obesity; we studied the following three major aspects: (1) changes in body composition and cardiometabolic risk, (2) changes in the adherence to a Mediterranean diet, and (3) changes in physical performance.

## 2. Measurements and Methods

### 2.1. Subject Recruitment and Research Context

A total number of 74 Caucasian children and adolescents subjects with overweight/obesity were recruited at the Healthy Lifestyle Institute of the University of Perugia (C.U.R.I.A.Mo., Centro Universitario Ricerca Interdipartimentale Attività Motoria) from March 2013 to December 2014. The total sample was divided into two subgroups by age: the children group (*n* = 43, age 5–12 yrs.) and the adolescent group (*n* = 31, age 13–17 yrs.). In the children's group (58.1% male and 41.9% female) there was a mean age of 9.79 ± 1.8 years (min 5–max 12), with no differences for gender, while in the adolescent group (38.7% male and 61.3% female) there was a mean group age of 14.32 ± 1.38 (min 13–max 17), with no differences for gender.

Children were evaluated at the Pediatric Clinic of the local hospital and those who met the inclusion criteria were referred to the Health Lifestyle Institute (C.U.R.I.A.Mo.).

At the first visit patients were assessed for the anthropometric values. Inclusion criteria for the enrolment were BMI over 85° percentile [42], the absence of contraindications to perform physical exercise, and parents' informed written consent to the lifestyle intervention. The intervention followed the C.U.R.I.A.Mo. lifestyle approach for children and adolescents (approved by the local Ethics Committee, CEAS, Umbria Region, HREC number 1/10/1633), a multidisciplinary structured program including the nutritional intervention, the exercise intervention, and the psychological intervention [32]. All participants followed the three different parts of the intervention described later. The 9% of the total sample recruited (4 children) did not start the program because they were still engaged in other sport activities or due to family difficulties in managing the timetable of the activities. Parents' work difficulties were cited as the main barriers to participation.

### 2.2. The Structured Intervention Programs

Subjects followed two different group programs on the basis of their group age. The duration of the intervention program was different for the two age-groups lasting 6 months for the children's group and 3 months for the adolescent group. Using a quasi-experimental study design children and adolescents were assessed before (*T*0) and at the end of the intervention (*T*1, six months for children and three months for adolescents).

The* nutritional intervention* was structured in individual counselling sessions and educational group sessions that involved all the family components. The counselling activity is not prescriptive in nature; it does not foresee the use of dietary requirements but give patients the tools (food diary) and nutritional information to verify the eating habits and promote the change of those unhealthy.

All the children family underwent to three individual nutritional counselling sessions of 30–60 min duration at the beginning, in the middle, and at the end of the program. During the intervention four nutritional education group sessions (based on an interactive method of teaching/learning) were organized for the parents (both mothers and fathers) of the children and the adolescents. In this phase the family (children/adolescents and their parents) became able to choose and eat healthy foods, through practical advice of the dietician.

During each meeting, the general principles of healthy eating were clarified to the children/adolescents and their parents, in order to explain strategies to reduce the high-energy food consumption and increase the consumption of vegetables, fish, fruits, and foods rich in fiber, improving the adherence to the principles of the Mediterranean diet.

These meetings should promote the restriction of extra energy from foods with saturated fats, sugars, and salt. Whole fresh fruit and vegetables daily-consumption should be promoted, and sugared beverages should be replaced by water.

All the advertisement follows the Italian Standard Treatments of Childhood Obesity (SIO) [16, 17, 43].

In addition, the adolescents attended four interactive sessions on healthy nutrition recommendations (maximum 6–8 subjects/group).

To assess the change in eating habits of patients it has been proposed to fill out a questionnaire (KIDMED) that allowed the calculation of the Mediterranean diet score.

The* exercise intervention* was planned with a duration of 6 months and a frequency of 2 sessions per week for children and with a duration of 3 months and a frequency of 2 sessions per week for adolescents. The exercise program aimed to improve both general physical fitness (including aerobic capacity, flexibility, and muscle strength) and the pleasure in leisure time physical activity. The activities for children were organized in groups of 6–12 participants. All the training sessions were supervised by two exercise physiologists and the activity consisted of four phases: warm-up (15 minutes), exercises to improve conditional and coordination skills (50 minutes), game group (15 minutes), and final stretching (10 minutes) for a total of 90 minutes per session. The training program for the adolescents (5-6 participants/group) was supervised by one exercise physiologist. Each session lasted 90 minutes and included 60 minutes of cardiovascular workouts plus 30 minutes of circuit training for muscular strength. The cardiovascular activity was performed using ergometers (Run 500, Runrace 1200 Hc, Bike Race, Recline 600 Xt Pro, and Top 600xt Pro and Synchro, Technogym, Cesena, Italy). The intensity of the exercises was gradually increased (5% every three weeks) starting from 50% of heart rate reserve (HRR) calculated by Karvonen's formula to reach 70% HRR [44]. Isotonic machines (Lat machine, chest press, leg press, and leg extension, Technogym, Cesena, Italy), free loads, and equipment were used for muscular strength workouts, with gradual increase from 55% up to 70–80% of 1 repetition maximum (RM), according to the adolescent's fitness level, estimated at the beginning of exercise with the equation of Brzycki [45].

The* psychological intervention* included an initial intensive phase (five sessions lasting one month and a half) characterized by a family-based approach involving both parents in a counselling focused on the needs of family.

In more detail the psychological intervention follows three phases: (1) psychological evaluation: initial intensive phase consisting of five meetings with the child/adolescent and parents, designed to evaluate a number of risk factors associated with overweight/obesity in families; (2) first phase of psychoeducational groups: four meetings involved both parents supporting the exercise and nutrition program; (3) second phase of psychoeducational groups: three meetings for teenagers (peer) focused on the impact of overweight/obesity, self-esteem, and body image. The goal was to share among peers (supported by a psychologist) the main risk factors associated with obesity developmental age. Four psychoeducational groups of 1 hour for the parents of all participants addressing the psychological determinants of obesity in the parental relationship, using a video-feedback approach [46], were also organized. Parents were trained by using video-feedback and self-reflection, to allow more insight and empathy, broadening their coping skills toward the use of healthy life habits. The objective of this intervention is to create a personalized program based on the characteristics of each household and parents, made up of meetings followed by children/adolescents and their parents.

### 2.3. Measurements

#### 2.3.1. Anthropometric Measurements

Height, body weight, body mass index (BMI), waist circumference (WC), waist-to-height ratio (WHTR) [47], and body composition were measured in all participants using standard techniques [48].

The waist-to-height ratio (WHTR), calculated by dividing WC by height, is an anthropometric index for measuring central adiposity and it is useful for identifying cardiometabolic risk. Childhood obesity is in fact linked to an increased prevalence of cardiovascular and metabolic diseases in adulthood [49, 50]. Body composition was measured using air displacement plethysmography (BOD POD; Life Measurement Inc., Concord, CA, USA) [51]. Body density was calculated as body mass divided by corrected body volume. Fat mass was calculated using the equation of Lochman [52] and fat-free mass subtracting fat mass from body weight. Of the total subjects recruited that completed the intervention, 6 children and 8 adolescents had no data on body composition because of the lack of the equipment (BOD POD) at the time of their enrolment.

#### 2.3.2. Nutrition Habits

Nutrition habits were assessed at the baseline and after the intervention for all participants, using the KIDMED self-report questionnaire [53]. The KIDMED is an index consisting of 16 items, based on principles sustaining Mediterranean dietary patterns (12 items) as well as those that undermine it (4 item). The range is from 0 to 12. It can be self-administered (adolescents) or conducted by interview (children with their parents). In the KIDMED questions denoting a negative connotation with respect to the Mediterranean diet are assigned a value of −1 and those with a positive aspect +1. The sums of the values are classified into three levels: (1) ≥8, optimal Mediterranean diet; (2) 4–7, improvement needed to adjust intake to Mediterranean patterns; (3) ≤3, very low diet quality.

#### 2.3.3. Physical Performance

The measurements were different for the two groups. For children (5–12 years) a set of functional motor fitness tests was performed (at sessions number 3 and number 5) to evaluate the* aerobic capacity* (endurance),* speed*,* flexibility,* and* dynamic muscle strength.* The motor fitness test set [54, 55] was performed individually and comprised: (a) 6 minutes' walking test; (b) 30 metres' speed test; (c) Sargent Test; (d) bending test.; (e) medicine ball throw [56–60].

- 6 minutes' walking test: this test was adopted to quantify functional exercise capacity. The distance that a children can walk as fast as possible in a period of 6 minutes was recorded.
- 30 m speed test: children had to run the distance of 30 metres, as quick as possible. The measurement of the test was made with a chronometer. It began simultaneously, with the start ordered by the exercise physiologist. Each performance's time was recorded in seconds and hundredths.
- Sargent Test: children with fingers painted with ink were invited to stand in front of a wall and to extend their arms as high as possible. The trace left on the wall was recorded by the exercise physiologist. Then, after bending their knees (90 degrees) the children were asked to execute a vertical jump. The difference in centimetres between the two signs (before and after jumping) was considered.
- Sit and reach test: a standard method to assess hip and trunk flexibility was executed from a standing position (vertical bending, VB) and a seated position (horizontal bending, HB) by using a sit and reach box of 40 cm × 40 cm (Technogym) and recording the distance reached by the tip of the fingers.
- Medicine ball throw: children were asked to sit behind the throwing line holding the ball with two hands stretched above the head and, after extension of the body backwards, to throw the ball as far as possible. Feet had to remain on the ground. The ball trajectory had to be vertical to the throwing line. Before carrying out the test the technique was demonstrated, with a particular emphasis on the throwing angle (30–40 degrees) and the synchronization of body and hands.

During the test the distance between the throwing line and the point where the ball fell onto the ground was recorded; the best performance from three attempts was evaluated.

For the adolescents functional tests were performed at the beginning and at the end of 24 exercise sessions (3 months, *T*1) to evaluate aerobic capacity (VO_2_ max), flexibility, and dynamic muscle strength. Aerobic capacity was measured using indirect calorimetry with a gas exchange analyzer (FitMate, Cosmed, Rome, Italy). The test was conducted using a Balke protocol [61] on a treadmill, modified according to the* FitMate* user manual [62]. To determine the dynamic force of arm flexor and extensor muscles and of leg extensor muscles, the indirect method of extrapolation to* one repetition max* was used [45]. Four isotonic machines were selected for the exercise program (leg extension and horizontal leg press for the leg,* pull down on the frontal plane *at the Lat machine, and* push movement on the transverse plane* at chest press machine, Technogym, Cesena, Italy). A maximum number of repetitions between 8 and 12 were considered. To determine the value of 1 RM the predicted* equation of Brzycki* (1 RM = lifted load × (1.0278 − 0.0278 × reps) − 1) was used [45, 63]. To assess hip and trunk flexibility a standard method of sit and reach test was executed from the standing position (vertical bending, VB) and in a seated position (horizontal bending, HB) by using a sit and reach box that measured 40 cm × 40 cm (Technogym); the distance reached by the tip of the fingers was recorded [64].

### 2.4. Statistical Analysis

The sample size was calculated based on WHTR endpoint as a main predictor of cardiometabolic risk. A sample size of 74 achieves 89% power to detect a mean of paired differences of 3% with an estimated standard deviation of differences of 8% and with a significance level (alpha) of 0,05 using a two-sided paired *t*-test.

Descriptive analysis in terms of mean, standard deviation, and percentages were computed for the variables investigated. Student's *t*-test for Paired Sample was used to compare all assessment measures (anthropometry, nutrition habits, and physical activity) before and after intervention (*T*0-*T*1). *t*-test effect sizes were also reported in terms of Cohen's *d*. According to the literature [65] effect size *d* = 0.2 is small, *d* = 0.5 is medium, and *d* = 0.8 is large.

Analyses were limited to participants with baseline data on the different measurements and performed using SPSS, version 21.0.

## 3. Results

### 3.1. Anthropometric Measurements

Anthropometric data at the baseline and after the intervention are reported in Table 1 for the children's group and in Table 2 for the adolescent group.

#### 3.1.1. Children

After the intervention (*T*1) data showed a significant decrease in all the measures. BMI (*t* = 3.96; *p* < .001; *d* = 0.27), waist circumference (*t* = 4.02; *p* < .001; *d* = 0.27), and WHTR index (*t* = 3.36; *p* < .001; *d* = 0.44) showed a significant reduction with a small effect size. Regarding body composition (subgroup of 33) data showed a significant decrease with a large effect size of percentage for fat body mass (*t* = 4.61; *p* < .001; *d* = 0.83) and a significant increase in percentage of fat-free mass with a medium effect size (*t* = −4.77; *p* < .001; *d* = 0.50).

#### 3.1.2. Adolescents

After the three months intervention adolescents showed a significant decrease in waist circumference (*t* = 5,60; *p* < .001; *d* = .35). The subgroup (*N* = 23) assessed with BOD POD showed a reduction of fat body mass percentage (*t* = 4.52; *p* < .001; *d* = 0,50) with a medium effect size and an increase of fat-free mass (*t* = −3.17; *p* = .004; *d* = .41).

### 3.2. Nutritional Habits Measure

#### 3.2.1. Children 5–12 Years

Children showed a significant improvement in KIDMED scores with a medium effect size (*t* = −3.33; *p* = .002; *d* = .59) from *T*0 = 6.73 ± 2.27 to *T*1 = 7.93 ± 1.74.

#### 3.2.2. Adolescents 13–17 Years

Adolescents showed a significant improvement in KIDMED scores with a large effect size (*t* = −5,94; *p* < .001; *d* = 1,08) from *T*0 = 5.68 ± 2.76 to *T*1  8.39 ± 2.42.

### 3.3. Physical Performance

#### 3.3.1. Children 5–12 Years

As reported in Table 3, the mean distance walked within six minutes increased (Δ = 37.03 ± 144.03) after three months of exercise, with no statistical difference between two times of evaluation.

Strength values were raised, as expected, in response to the progressive work load proposed during the exercise period. The data showed a significant increase with a medium effect size in ball throw (*t* = −4.24; *p* < .001; *d* = 0.73 and *t* = −5.53; *p* < .001; *d* = 0.77). The sprint time of the 30 m test improved significantly, with a large effect size (*t* = 8.29; *p* < .001; *d* = 1.3). Finally, flexibility improved only the bending test results from the seating position (*t* = −2.01; *p* = .05; *d* = 0.20) with a small effect size.

#### 3.3.2. Adolescents

As shown in Table 4, maximal oxygen consumption (VO_2_ max) increased (Δ = .48 ± 4.53) after three months of exercise, with no statistical difference between two tests. In strength values there was a significant increase with a large effect size in every exercise at the isotonic machine (except in a leg press test that presented *t* = −7.15; *p* < .001; *d* = .71), while the flexibility value improved only in bending test from standing position with a small effect size (*t* = −2.38; *p* = .025; *d* = .23).

## 4. Discussion 

The aim of the present study was to investigate the effects of a multidisciplinary family-based lifestyle intervention to treat overweight/obese children and adolescents.

The first results of our structured intervention demonstrate effectiveness in reducing the cardiometabolic risk through a significant reduction of WHTR and changes in body composition.

The structured multidisciplinary intervention shows changes in nutritional habits (greater adherence to Mediterranean diet) and improvements in physical performance.

The model consists in a multidisciplinary approach focused on family's involvement and based on the best practices of childhood obesity treatment [18, 23, 24, 37, 39].

Evidence suggests that a multidisciplinary approach is the most effective strategy to promote lifestyle changes and confirms that family involvement and peer support with group meetings are the key to ensure adherence to the healthy path [35, 36, 38]. In line with previous evidence, the results of this study seem to be able to show how a multidisciplinary approach based on the family is effective not only in children but also in the adolescent group, where a significant decrease in waist circumference (*p* < .001), a significant reduction of fat body mass percentage (*p* < .001), and a significant decrease in waist circumference (*p* < .001) and in fat body mass percentage (*p* < .001) were observed as well as improvement of the nutritional habits (*p* < .001) and strength parameters (Table 4).

In particular it is suggested that adolescent family involvement and peer support with group meetings are the key to ensure adherence to the path and promote the lifestyle change: during the physiological intervention three meetings for teenagers (peer) focused on the impact of overweight/obesity, self-esteem, and body image were expected to share among peers (supported by a psychologist) the main risk factors associated with obesity developmental age [18, 39–41]. Further studies should better investigate the contribution of such kind of support in the different age-group populations.

Furthermore evidence shows that adherence to physical activity is poor when only children and adolescent are involved without their families or friends [28, 29, 66] and that restricting diet causes damaging effects on health and it is not effective in the long term [18, 67].

Multicomponent approach structured in an intervention model including simultaneously nutritional aspects, physical activity, sedentary time reduction, and behavioral changes can deliver better outcomes than isolated interventions [18, 39]. The inclusion of the family in children's treatment programs helps children and parents to overcome barriers of lifestyle and behavioral changes [29] treatment is optimized if family members are specifically targeted in treatment.

PA attitudes are influenced by individual, social, environmental, and community aspects [68].

Family influences and friend support can act in improving physical activity habits [69–71]. Like reported by Salvy et al. [72, 73] children and adolescents are more physically active when in the presence of peers and it is likely that these positive feelings increase the enjoyment and youth motivation to engage in physical activity (PA) [74].

Efficacy expectations are related to different psychosocial responses [75]. The literature showed that self-efficacy and family support are associated with the pleasure in physical activity; furthermore self-efficacy can act as both a determinant and a consequence of physical activity participation, both in children and in adolescents [70]. Also goals and friend support are important to favour the participation in PA in youth groups [72, 73]. In our experience the peer support during all the group activities proposed is one of the keys to overcome children limits to move.

The present results confirm that this strategy is effective in ameliorating, in the short term (3–6 months), the health status, the nutrition habits, and the physical performance of children and adolescents. In particular, the present data demonstrate that after the intervention the participants significantly reduced BMI, WC, WHTR, and fat mass and improved fat-free mass, adherence to the Mediterranean diet, and physical fitness. The positive results of the anthropometrics measurements in terms of a reduction of visceral fat, supported by the concomitant reduction in waist circumference and body fat mass, suggest that the intervention can reduce the cardiovascular risk factors already demonstrated in children and adolescents [76, 77]. In more detail the WHTR index is linked to cardiovascular risk also in children and adolescents population [49, 50]. Santoro et al. (2013) [78] reported that a WHTR higher than 0.60 is associated with a statistically significant increased risk of metabolic syndrome, prediabetes, hypertension, and dyslipidemia. In our sample, despite a statistically significant variation of values, the risk remains high. The improvement in body composition obtained with the intervention is noteworthy because in children after 6 months the fat mass was reduced by about 6% and fat-free mass increased by about 3%, while in adolescents after only 3 months the reduction in fat mass was about 4% and the increase in fat-free mass was about 3%. The measurement of these differences in body composition is reliable and accurate because it was performed using plethysmography with BOD POD, a gold standard method to evaluate body composition in children [51]. This means that during the growing age the body is particularly sensitive to dietary and physical activity changes and that lifestyle interventions in this period can give optimal results.

In order to evaluate the nutrition habits of the participants we have chosen the KIDMED questionnaire that offers a simple Mediterranean diet adherence score, particularly appropriate in a Mediterranean country like Italy [53]. It is well established that the Mediterranean diet has an important role in prevention of cardiovascular diseases and diabetes and has a beneficial effect in the treatment of the metabolic syndrome and of obesity [79, 80]. In this intervention for children, we combined the educational nutrition groups for the parents with three individual nutritional visits with both the child and his/her parents to raise their awareness about the importance of the Mediterranean diet and the consumption of healthy foods in the whole family. The family-based approach augments the possibilities of improving the quality of the nutrition because the children, who are capable of internalizing what they learn, become positive leaders and an example in establishing a healthy lifestyle in the whole family [81]. Through family meals, children can eat more whole grains, fruits, and vegetables and consume less unhealthy fats. Parents should involve kids in preparing food to make a positive effect on their attitudes toward obesity prevention. For the adolescents, four educational nutrition groups were included and dedicated directly to them, using a proper interaction and language. As for the children, four specific education groups were delivered only to their parents. The final result of the nutritional intervention is positive for both groups and in particular in adolescents, where the Mediterranean score increased by about 30%. It must be stressed that in literature Mediterranean diet adherence has been inversely associated with change in BMI among adolescents [82–84]. Finally, it should be stressed that the nutrition intervention improved both children's and parents' nutrition habits [85]. The study confirms the importance of a family-based approach for childhood obesity, which is superior to the conventional approaches or programs consisting only of food restriction [29, 81, 85]. Physical activity and the avoidance of sedentary habits played an important role through the improvement in body composition and reduction in weight [86, 87]. In its recommendations WHO indicates that children and young people aged 5–17 years should accumulate at least 60 minutes of physical activity every day [88]. At baseline, the participants in the study did not follow this recommendation. The exercise intervention aimed to improve general physical fitness and the pleasure in physical activity, teaching the whole family the motor and social skills needed to improve the physical activity levels of the children and adolescents.

In regard to the type of movement, WHO recommends that most of daily physical activity should be aerobic, including moderate to vigorous activity that strengthens muscle and bone; to support the participation all activities can be performed as part of playing motor games (running or jumping is optimal like bone-loading). Katch [89] examined the exercise-induced changes in muscular and cardiovascular function during the pre- and the postpubertal age. He underlined that during prepuberty it is possible to observe small training-induced biological alterations because of the lack of hormonal drive. For these reasons it is recommended that workouts should aim toward skill acquisition rather than physiological conditioning. In contrast, for the adolescents exercise-induced conditioning changes are well documented in literature [90]. According to Kramer et al., in our intervention children and adolescents were involved in non-high-intensity efforts [91]. In particular for the activity group of the adolescents, exercises with isotonic machines and low free weights were chosen and these involved the bigger muscular groups, thus catering for the abilities of everyone. In this intervention, a circuit training program was proposed to obtain possible cardiorespiratory benefits and improve on bone health and cardiovascular and metabolic health biomarkers. However, in children, to encourage participation and increase the fun, short bouts of high intensity activity were introduced, alternated with low/moderate intensity physical activity, and group activity [92].

As shown previously in Tables 3 and 4 improvements in (1) dynamic strength; (2) cardiorespiratory efficiency; (3) the speed of the children, and (4) flexibility were observed. In addition, to train aerobic capacity and flexibility resistance training was also included in the exercise intervention. In adolescents cardiovascular activity was presented using ergometers and with gradually increasing work intensity (5% every two weeks) from 50% up to 70–80% of heart rate reserve [44] combined with free loads and work at isotonic machines with a gradual increase from 55% up to 70–80% of 1 repetition maximum (RM), according to an adolescent's basal fitness level. After three months of exercise, data showed that strength values increased, as expected, in response to gradually augmented load used during exercise periods. In particular, relevant changes in the dynamic strength of the upper limbs and trunk and lower extremity strength were observed, with a medium and large effect size.

In adolescents, we tested the effects of the intervention on VO_2_ max and did not observe significant changes. It is likely that the absence of improvement in aerobic power is related to the fact that we used workloads at a lower intensity than that required to obtain significant increases in adaptations in cardiovascular fitness.

As regards flexibility, the existing studies confirm a role for genetic influences on the individual differences but estimates vary widely. 18–55% of the variation in flexibility (as measured by the sit and reach test) in children and young adults could be explained by genetic influences (Chatterjee and Das 1995; Maes et al. 1996; Okuda et al. 2005) [93–95]. In addition, the study by Maes et al. 1996 detected significant shared environmental influences on flexibility [94].

In the groups, we noticed few improvements in flexibility values, estimated by the seated and standing sit and reach test. Children increased their flexibility from a seated position, while adolescents improved their flexibility from a vertical standing position; in both evaluations we have a small effects size [93–95].

## 5. Conclusions

This study reports the first results of an original multidisciplinary lifestyle intervention based on a family-based approach, demonstrating that such kind of approach allows obtaining positive results in lifestyle habits changing in not only children but also adolescents groups with overweight or obesity, after a short period (3 to 6 months). These first results are promising in order to facilitate the initiation and adherence on a physical activity program; the participants improved health related outcomes. These first results confirm the statement of the American Dietetic Association (ADA) that underlines the effectiveness of multicomponent programs including behavioral counselling, promotion of physical activity, parent training/modeling, dietary counselling, and nutrition education [96]. These first results are encouraging regarding the effectiveness of these programs in counteracting overweight/obesity and bad lifestyle habits of children and adolescents to decrease cardiometabolic risk, but they are not conclusive. It remains to demonstrate the long term efficacy of the intervention and the methodological limitation of the present study that lacks a control group which was not included for ethical reasons must be stressed.

## Figures and Tables

**Table 1:** *Children's data*. Descriptive, Paired Sample *t*-test of the anthropometric measure at *T*0-*T*1. Data are presented as mean ± SD.

Characteristics	*T*0	*T*1	*t*	*p*	*Cohen's d*
*Anthropometric data*					
Children (5–12)					
BMI (kg/m^2^)	27.62 ± 3.50	26.71 ± 3.18	3.96	.001	.27
WC (cm)	91.82 ± 10.20	89.23 ± 9.24	4.02	.001	.27
WTHR (cm)	0.63 ± 0.05	0.61 ± 0.04	3.36	.002	.44
FM (kg)	24.37 ± 8.51	21.96 ± 8.38	3.03	.005	.28
FM (%)	41.21 ± 8.06	35.34 ± 5.82	4.61	.001	.83
FFM (kg)	35.86 ± 6.69	38.72 ± 7.97	−3.86	.001	.39
FFM (%)	61.60 ± 5.70	64.53 ± 6.05	−4.77	.001	.50

Statistical significance was considered at *p* < .05; BMI = Body mass index; WC = waist circumference; WHTR = waist to height ratio; FM = fat body mass; FFM = fat-free mass.

**Table 2:** *Adolescent's data*. Descriptive, Paired Sample *t*-test of the anthropometric measure at *T*0 and *T*1. Data are presented as mean ± SD.

Characteristics	*T*0	*T*1	*t*	*p*	*Cohen's d*
*Anthropometric data*					
Adolescents (13–17)					
BMI (kg/m^2^)	32.50 ± 5.30	31.43 ± 5.76	5.20	.001	.19
WC (cm)	106.22 ± 12.52	102.00 ± 11.66	5.60	.001	.35
WHTR (cm)	0.65 ± 0.09	0.63 ± 0.08	2.68	.012	.23
FM (kg)	36.55 ± 11.24	33.38 ± 12.38	4.76	.001	.27
FM (%)	40.55 ± 6.86	36.98 ± 7.38	4.52	.001	.50
FFM (kg)	52.97 ± 7.13	54.83 ± 8.57	−2.14	.044	.24
FFM (%)	59.79 ± 8.25	63.01 ± 7.38	−3.17	.004	.41

Statistical significance was considered at *p* < .05; BMI = body mass index; WC = waist circumference; WHTR = waist to height ratio; FM = fat body mass; FFM = fat-free mass.

**Table 3:** *Children's data*. Descriptive, Paired Sample *t*-test of the physical activity measurement at *T*0 and *T*1. Data are presented as mean ± SD.

Characteristics	*T*0	*T*1	*t*	*p*	*Cohen's d*
*Physical activity data*					
Children (5–12)					
6 MinWT (cm)	679.54 ± 85.66	716.56 ± 159.12	n.s.	n.s.	n.s.
Ball TA (cm)	4.47 ± 0.77	5.06 ± 0.97	−4.75	.001	.67
Ball TB (cm)	4.74 ± 1.10	5.55 ± 1.42	−6.71	.001	.63
30 m sprint (s)	7.24 ± 0.79	6.37 ± 0.79	7.98	.001	1.1
VB (cm)	−3.05 ± 8.45	−2.10 ± 8.70	n.s.	n.s.	n.s.
HB (cm)	32.26 ± 9.97	33.38 ± 9.72	−2.06	.05	.11
Sargent (cm)	23.03 ± 5.99	26.60 ± 7.07	−4.66	.001	.54

Statistical significance was considered at *p* < .05; 6 MinWT = six minutes' walking test; ball TA = medicine ball throw ahead; ball TB = medicine ball throw behind; 30 m sprint = 30 metres' speed test; VB = vertical bending value at sit and reach test, HB = horizontal bending values at sit and reach test; Sargent = Sargent Test Value.

**Table 4:** Descriptive, Paired Sample *t*-test of the physical activity measure of the adolescents (13–17) at *T*0 and *T*1 of the intervention. Data are presented as mean ± SD.

Characteristics	*T*0	*T*1	*t*	*p*	*Cohen's d*
*Physical activity data*					
Adolescents (13–17)					
VO_2_ max (ml/kg/min)	31.11 ± 7.64	31.60 ± 7.05	n.s	n.s	n.s
Lat (kg)	36.36 ± 8.61	44.03 ± 9.1	−11.00	.000	.86
Chest (kg)	30.5 ± 8.64	39.00 ± 10.64	−9.34	.000	.88
Press (kg)	175.29 ± 56.13	216.76 ± 60.33	−7.15	.000	.71
Lext (kg)	40.11 ± 12.52	52.68 ± 11.50	−7.09	.000	1.04
VB (cm)	−6.33 ± 8.97	−4.26 ± 9.30	−2.38	.025	.23
HB (cm)	30.19 ± 9.16	31.00 ± 9.50	n.s	n.s	n.s

Statistical significance was considered at *p* < .05; VO_2_ max = maximum rate of oxygen (O_2_) consumption; Lat = Lat machine test value; chest = chest press test value; press = leg press test value; Lext = leg extension test value; VB = vertical bending value at sit and reach test, HB = horizontal bending values at sit and reach test.
